# Proteomics analysis reveals a dynamic diurnal pattern of photosynthesis-related pathways in maize leaves

**DOI:** 10.1371/journal.pone.0180670

**Published:** 2017-07-21

**Authors:** Dan Feng, Yanwei Wang, Tiegang Lu, Zhiguo Zhang, Xiao Han

**Affiliations:** Biotechnology Research Institute, Chinese Academy of Agricultural Sciences, Beijing, P. R. China; University of the Sunshine Coast, AUSTRALIA

## Abstract

Plant leaves exhibit differentiated patterns of photosynthesis rates under diurnal light regulation. Maize leaves show a single-peak pattern without photoinhibition at midday when the light intensity is maximized. This mechanism contributes to highly efficient photosynthesis in maize leaves. To understand the molecular basis of this process, an isobaric tag for relative and absolute quantitation (iTRAQ)-based proteomics analysis was performed to reveal the dynamic pattern of proteins related to photosynthetic reactions. Steady, single-peak and double-peak protein expression patterns were discovered in maize leaves, and antenna proteins in these leaves displayed a steady pattern. In contrast, the photosystem, carbon fixation and citrate pathways were highly controlled by diurnal light intensity. Most enzymes in the limiting steps of these pathways were major sites of regulation. Thus, maize leaves optimize photosynthesis and carbon fixation outside of light harvesting to adapt to the changes in diurnal light intensity at the protein level.

## Introduction

Photosynthesis is the most important process for generating energy and supplying organic materials to plants. During this process, light energy is converted to chemical energy, and carbon from CO_2_ is incorporated into organic molecules in plant leaves. These processes support nearly all living organisms on Earth [1]. In diurnal cycling regulation, light intensity is the most important environmental factor impacting the photosynthesis rate [2–4]. Plant leaves have developed mechanisms to adapt to variations in light intensity caused by environmental conditions [5–7]. Over a certain range, the photosynthesis rate is positively correlated with light intensity [8–10]. Once the photosynthetic rate is maximized, plants cannot adapt to increased light intensity, and thus, the rate can decrease because of light damage under strong intensity illumination [11, 12]. To improve crop production, research has focused on photosynthesis as the basic production process of bioenergy and organic materials [13]. Studies of various crops have revealed different patterns of photosynthesis rates that are dependent on the pattern of light intensity during the diurnal cycle [11, 14]. In general, the diurnal patterns of the photosynthetic rate in crops can be classified into two categories: a single-peaked curve, such as in *Zea mays* [15, 16] and *Sorghum bicolor*, and a double-peaked curve, such as *Oryza sativa*. Some crops, such as *Glycine max*, exhibit different diurnal patterns among different cultivars. Maize, in which the photosynthetic rate shows a single-peaked curve, can endure high-intensity light at midday, and the diurnal pattern of its photosynthetic rate matches the curve of diurnal light intensity with a slight delay. Rice [17], which has a double-peaked curve, suffers from photoinhibition at midday, when high light intensity causes stomata closure and low efficiency in the chloroplasts. Hence, maize has a higher photosynthetic rate than rice [1, 18–21]. Consequently, it is widely accepted that efficient photosynthesis in maize leaves contributes to this plant’s high productivity [22, 23]. To understand the molecular mechanism involved in the diurnal regulation of the photosynthetic rate in maize, several studies have been conducted on gene regulation, photosynthetic enzyme activity and photosynthate partitioning [24–26]. The results revealed that the diurnal regulation of the photosynthetic rate requires delicate control of the genetic composition, gene expression, protein levels, protein modification and metabolic transport related to photosynthesis [27].

Recently, a substantial amount of genome information from several crops have become available due to advancements in sequencing technology. The genetic information of rice, maize, millet and sorghum could improve our knowledge of the physiological activities associated with photosynthesis in crops [28–32]. The transcriptome of Arabidopsis revealed that diurnal cycle regulation is a major regulatory mechanism in Arabidopsis gene expression; indeed, up to 50% of gene expression is correlated with diurnal rhythms [5]. Several pathways are involved in this regulation, including carbon and nitrogen metabolism pathways, the isoprenoid biosynthetic pathway and hormone regulation [5, 28]. In rice, gene expression is also controlled by diurnal regulation. An important component of the light-harvesting complex of photosystem II—the chlorophyll a/b binding protein—was identified as a circadian rhythm indicator. Additionally, phytochrome photoreceptors (PHYs) were characterized as regulators of the Dof transcription factor with circadian expression [33]. A dynamic proteomics analysis of rice leaves revealed the diurnal regulation of diverse biological processes, including energy conversion, photosynthesis, photorespiration, redox homeostasis and carbon and nitrogen metabolism, suggesting that these pathways could be the molecular bases of the diurnal regulation metabolism in rice [34]. However, these studies involved plants with double-peaked photosynthetic rate curves. Comparatively little is known about diurnal regulation on the genome level in crops with single-peaked curves, which utilize relatively delicate mechanisms to perform highly efficient photosynthesis. Research into the transcriptomes of maize and sorghum using different parts of leaves showed that site-regulated photosynthesis in these crops followed a single-peaked photosynthetic rate curve [35, 36]. Additionally, according to a microarray analysis with maize probes, 12% of all genes were found to exhibit clear-cut diel rhythm patterns of expression in leaves. Similar results were found for the aerial portion of maize, in which 10% of the examined genes displayed diel rhythm patterns of expression. However, the diurnal regulation of maize leaf proteins at the genome level remains elusive. Proteomic mass spectrometry (MS) is a powerful tool for quantifying changes in global protein expression patterns [37]. To understand the dynamic patterns of maize leaf proteins under diurnal light regulation, we performed an isobaric tags for relative and absolute quantitation (iTRAQ)-based quantitative proteomics study using the flag leaves of maize. The results revealed the possible molecular bases for photosynthesis with a diurnal single-peak pattern.

## Materials and methods

### Plant material

The maize (*Z*. *mays* L. ‘B 73’) used here was planted and grown under natural conditions in the field. The samples were collected from our field station where is normal experiment field for Chinese Academey of Argriculture without any endangered or protected species. We do not need any specific permissions for our experiments. At the filling stage, bracts were collected at 6:00, 9:00, 11:00, 12:00, 13:00, 14:00, 16:00 and 18:00 and weighed. The collected samples were immediately frozen in liquid nitrogen and stored at −80°C.

### Protein preparation

Leaves (1.0 g) were ground to a fine powder in liquid nitrogen and suspended in 4 ml of pre-cooled extract buffer (150-mM NaCl, 1% TrixonX-100, 1-mM dithiothreitol [DTT], 1-mM phenylmethane sulfonyl fluoride [PMSF], 50-mM Tris, pH 8.0) at 4°C for 2 h. The supernatant was collected by centrifugation at 400×g for 20 min at 4°C (SORVALL, Germany). The pellet was suspended in two volumes of 0.2-M CaCl_2_ solution and sonicated (1 min × 10 cycles). After incubating for 2 h at 4°C with stirring, the mixture was centrifuged at 15,000×g for 5 min, and the supernatant was collected. The combined supernatant was incubated with four volumes of cold acetone at –20°C for 2 h to precipitate proteins and then centrifuged at 15,000×g for 15 min at 4°C. The resultant pellet was air-dried to remove the residual acetone and dissolved in dissolution buffer (50% triethylammonium bicarbonate).

### Protein digestion

Protein reduction and cysteine blocking were performed. DTT was added to the protein extracts at a final concentration of 8 mM. The mixture was heated at 55°C for 25 min to break the disulfide bonds. After cooling the mixture, iodoacetamide (IAM) was added to a final concentration of 50 mM. The tube was then incubated at room temperature for 30 min in the dark. The mixture was centrifuged at 17,000×g for 10 min, and the supernatant was collected. After quantification by bicinchoninic acid assay (BCA), protein digestion was performed according to the filter-aided sample preparation (FASP) procedure. Briefly, 120 μg of protein from each sample was incorporated into an ultrafiltration tube (Millipore Microcon units, 500 μl, 10 kDa) and centrifuged at 14,000×g for 40 min. The ultrafiltration was repeated using 400 μl of DS buffer (100-mM triethylammonium bicarbonate) to remove DTT and other low-molecular weight components. Finally, trypsin (Promega, USA) was added at an enzyme-to-substrate ratio of 1:100. The preparation was incubated at 37°C overnight, and the resulting peptides were collected as a filtrate.

### iTRAQ labelling

The iTRAQ Reagents 8-plex kit (Applied Biosystems, CA) was used according to the manufacturer’s instructions, with slight modifications. For labelling, each vial of the iTRAQ Reagents 8-plex kit was allowed to come to room temperature. Then, each tube was spun to bring the solution to the bottom of the vial. A 50-μl volume of isopropanol was added to each room-temperature vial. Each vial was vortexed and then spun. The contents of each freshly prepared iTRAQ Reagents 8-plex vial were transferred to an individual sample tube, and each tube was vortexed and then spun. The pH was tested, and up to 5 μl of dissolution buffer was added to adjust the pH to between 7.5 and 8.5. The tubes were incubated at 25°C for 4 h. The contents of each iTRAQ Reagent8-plex-labelled sample tube were transferred into one tube, and the tube was vortexed and then spun. The resulting peptides were lyophilized to dryness, desalted with C18 cartridges (Sep-Pak Vac 3cc, Waters) and concentrated by vacuum centrifugation before the next step. The correspondence between the samples and the labels is as follows: 6:00 –tag 113, 9:00 –tag 114, 11:00 –tag 115, 12:00 –tag 116, 13:00 –tag 117, 14:00 –tag 118, 16:00 –tag 119 and 18:00 –tag 121.

### High-pH reverse-phase separation

The iTRAQ-labelled peptides were reconstituted in mobile phase A (10% acetonitrile [can], pH = 10.0, adjusted with ammonium hydroxide); mobile phase B was 95% ACN (pH = 10, adjusted with ammonium hydroxide). The solution obtained above was loaded onto the Agilent 1260 Infinity LC system (Agilent Technologies, Santa Clara, CA) with a C18 reverse-phase column (Durashell C18 (L), 5 μm, 4.6×250 mm, Agela Technologies, Tianjin, China). The peptides were eluted at a flow rate of 0.7 ml/min with a mobile phase B gradient of 40–90% from 0 min to 15 min, 90% to 97% from 15 min to 25 min, 97% from 25 min to 35 min, and 97% to 30% from 35 min to 40 min. The column temperature was 45°C. Finally, 94 fractions were collected, and any two fractions with the same time interval were pooled to reduce the fraction numbers (e.g., 1 and 48 and 2 and 49). The 23 resulting fractions were dried in a vacuum concentrator for further use.

### Low-pH nanoliquid chromatography tandem MS (nano-LC-MS/MS) analysis

The components were resuspended in 16 μL of solution C (0.1% formic acid in water) and then subjected to nano-LC and online electrospray MS/MS. The instrument used was an EASY-nLC1000 system (Thermo Fisher Scientific, Bremen, Germany) connected to a quadrupole-Orbitrap MS (Q-Exactive Plus) (Thermo Fisher Scientific, Bremen, Germany) equipped with an online nanoelectrospray ion source. Each 2-μL peptide sample was loaded onto the trap column (Thermo Scientific Acclaim PepMap C18, 100 μm × 2 cm) at a flow rate of 10 μL/min and subsequently separated on an analytical column (Acclaim PepMap C18, 75 μm × 15 cm) with a linear gradient from 2% D to 80% solution D in 76 min (0.1% formic acid in ACN). The flow rate of the column was 300 nL/min, the column temperature was 40°C, and the electrospray voltage applied at the inlet of the MS was 2.2 kV.

The Q-Exactive Plus MS was used in a data-dependent mode when switching automatically between MS and MS/MS acquisition. A survey of full-scan MS spectra (m/z 300–1500) was acquired with a mass resolution of 70 K, followed by 20 sequential high-energy collisional dissociation (HCD) MS/MS scans with a resolution of 17.5 K. One microscan cycle was recorded with a dynamic exclusion of 20 s.

### iTRAQ data analysis

All raw files generated by the Q-Exactive Plus instrument were searched using Thermo Proteome Discoverer (Thermo Fisher Scientific, Bremen, Germany; version 2.1.0.81) against a *Z*. *mays* database provided by The Universal Protein Resource (http://www.uniprot.org/uniprot, released 2012-11-01, with 43,971 entries). The enzyme specificity of trypsin was used with a maximal allowance of up to two missed cleavages for protease digestion. A parent ion tolerance of 10 parts per million (ppm) and a fragment ion mass tolerance of 0.02 Da were permitted by the Thermo Proteome Discoverer. The carbamidomethylation of cysteine and iTRAQ modification of peptide N-termini and lysine residues were set as fixed modifications, whereas the oxidation of methionine and iTRAQ8-plex labelling of tyrosine were specified as variable modifications.

A decoy database search strategy was adopted to estimate the false discovery rate (FDR) for peptide identification. In our study, proteins were assembled using a parsimony method and accepted if the Peptide FDR<1% and if the protein probability exceeded 99.0%. Proteins containing similar peptides that could not be distinguished based on MS/MS analysis alone were grouped to satisfy the principles of parsimony. One protein containing at least two unique peptides were identified. Proteins with 1.5-fold changes were considered differentially abundant proteins (S1 Table). The raw data accompanying this study have been deposited into the iProX with the identifier IPX00085001.

### Protein mass spectrum hierarchical cluster analysis and statistical analysis

To cluster the maize leaf proteins of the eight time points, we performed hierarchical and k-mean cluster techniques. The heatmap.2 function of the R package g plots was used to produce the graphical visualization of the dendrogram. We used Pearson’s correlation coefficient and scatterplots to determine the relationship between two random samples at different time points. Several CRAN packages were applied for the statistical analyses in the R environment (http://cran.r-project.org/). The proteins were categorized by the Gene Ontology (GO) David Tool, which can explain biological processes, molecular functions and cellular components.

### RNA isolation

Total RNA was extracted using TRIzol Reagent (Life Technologies) following the manufacturer’s instructions with slight modifications. Approximately 0.1 g of frozen leaf was ground to a fine powder with a steel ball and liquid nitrogen in a 2-ml tube. Then, 1 ml of TRIzol Reagent was added, and the mixture was violently shocked. Subsequently, 200 μL of chloroform was added and mixed vigorously. The mixture was centrifuged at 12,000 rpm for 10 min at 4°C. The supernatant was mixed with 500 μL of TRIzol and 200 μL of chloroform and centrifuged at 12,000 rpm for 10 min at 4°C. The supernatant was mixed with 500 μL of isopropanol, incubated for 15 min at 4°C, and then centrifuged at 12,000 rpm for 10 min at 4°C. The precipitate was washed with 75% ethanol. The precipitate was air-dried and dissolved in RNase-free water. The RNA integrity was assessed by agarose gel electrophoresis.

### cDNA synthesis and quantitative reverse-transcription polymerase chain reaction (qRT-PCR) analysis

Up to 1 μg of total RNA was used for cDNA synthesis using the First Strand cDNA Synthesis Kit (TOYOBO) according to the manufacturer’s instructions. qRT-PCR was performed using the TransStart Top Green qPCR SuperMix (Transgen Biotech) according to the manufacturer's instructions and the iQTM5 Multicolour Real-Time PCR Detection System (BIO-RAD). The qRT-PCR reaction conditions were as follows: 95°C for 3 min; 40 cycles at 95°C for 10 s and 60°C for 20 s and 72°C for 30 s and 80°C for 10 s, 72°C for 5 min; and a final increase of 0.5°C every 10 s from 55°C to 95°C. At each time point, three technical replicates were performed. The relative changes in the gene expression levels were calculated using the 2^−ΔΔCt^ method. The primers used were designed by DNAMAN (S2 Table). The actin gene was used as a reference gene.

## Results

### Protein identification

During the diurnal cycle, at the filling stage, maize flag leaves were collected at eight time points for iTRAQ proteomics analysis, and a total of 1,749 proteins were identified. Among these proteins, 73 were larger than 90 kDa, 335 were between 50 and 90 kDa, 1,011 were between 20 and 50 kDa, and 330 were between 0 and 20 kDa. For protein identification, the coverage indicates the percentage of peptide covering protein sequence. The average coverage of the peptide sequences was 11.4%. There were 38 proteins with more than 10 unique identified peptides and 749 with 3–9 unique identified peptides; the remaining proteins had one or two peptides.

**Fig 1:**
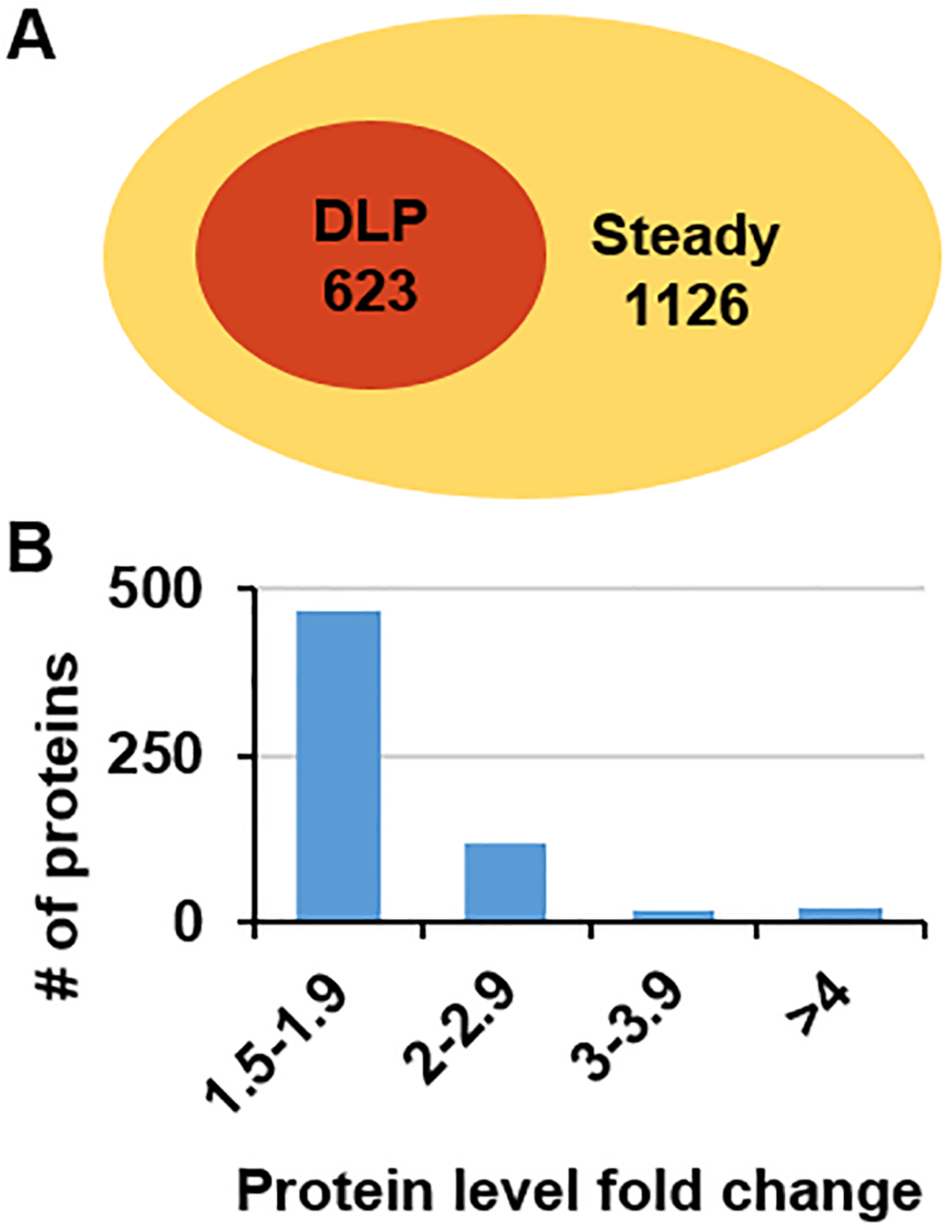
Generation of iTRAQ proteomics data. (A) Venn diagram of identified proteins. The numbers indicate the counts of identified maize leaf proteins with differential and steady levels. (B) The distribution of the fold-changes of the identified proteins.

The quantitative levels of the proteins at the eight time points were analysed by comparing their maximum and minimum levels. In total, 1,126 proteins exhibited steady levels at all eight time points, whereas 623 proteins exhibited differential expression, with a fold change of more than 1.5 between the maximum and minimum values at one or more time points (Fig 1A and 1B and S3 Table). Subsequent bioinformatics analyses focused on these differentially expressed proteins.

### Functional annotation of the identified proteins

**Fig 2:**
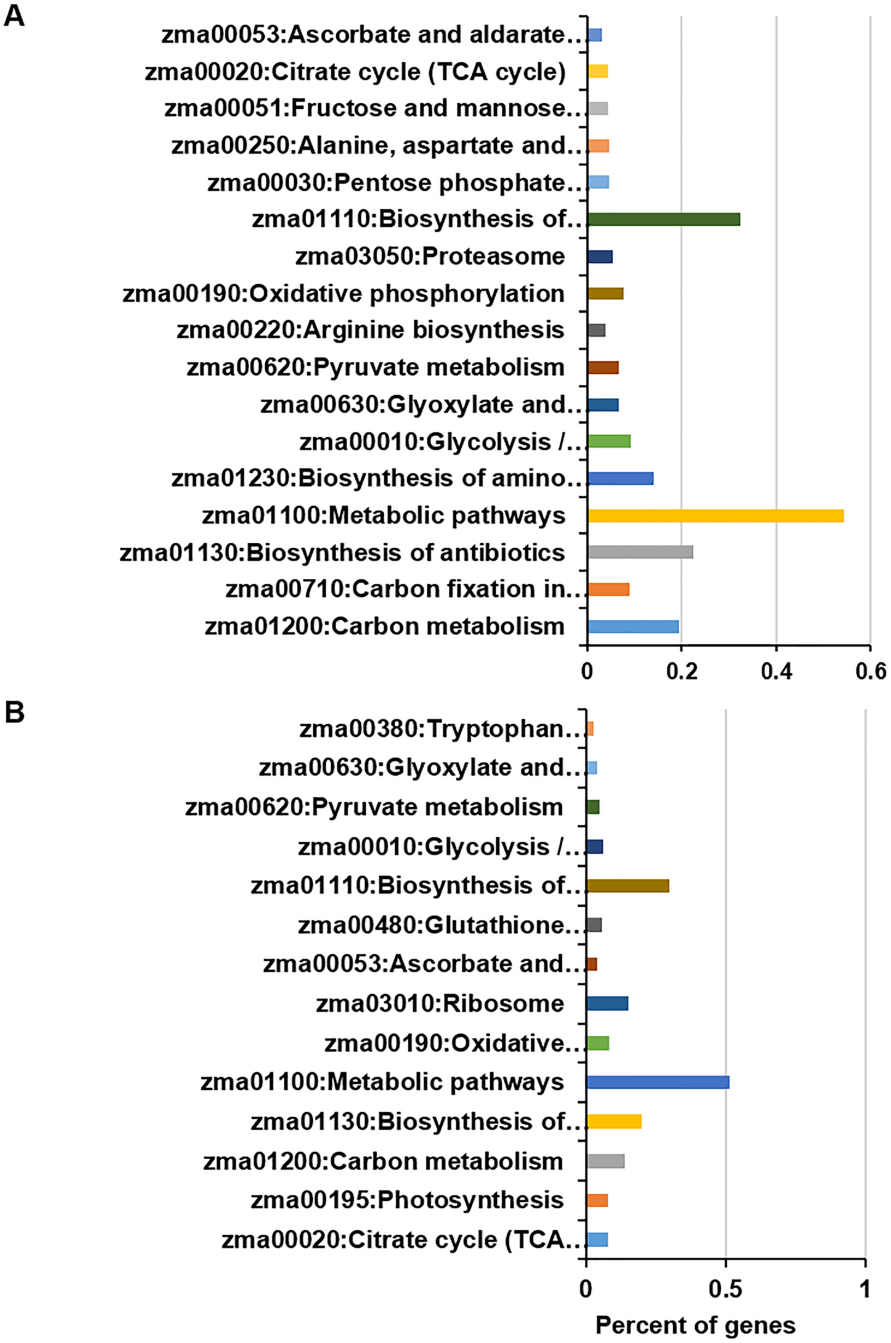
KEGG pathway analysis of the identified proteins. (A) Identified proteins with steady levels are enriched in diverse KEGG pathways. (B) The most significantly enriched pathways corresponding to the identified proteins exhibiting differential levels.

According to the Kyoto Encyclopedia of Genes and Genomes (KEGG)-annotated maize genome, the steady proteins in this study are widely spread across diverse biological processes, with approximately 54% of the identified proteins involved in metabolic pathways (Fig 2A). The top-enriched KEGG pathways (P<10E-4) were carbon metabolism; carbon fixation in photosynthetic organisms; the biosynthesis of antibiotics; metabolic pathways; the biosynthesis of amino acids; glycolysis/gluconeogenesis; glyoxylate and dicarboxylate metabolism; pyruvate metabolism; arginine biosynthesis; oxidative phosphorylation; the proteasome; the biosynthesis of secondary metabolites; the pentose phosphate pathway; alanine, aspartate and glutamate metabolism; fructose and mannose metabolism; the citrate cycle (tricarboxylic acid [TCA] cycle); and ascorbate and aldarate metabolism. In addition, compared with the annotated proteins in the whole genome, carbon metabolism, biosynthesis of antibiotics, biosynthesis of secondary metabolites and metabolic pathways were detected for more than 10%.

Differentially expressed protein identified via diurnal dynamic proteomics were also involved in diverse biological processes, including the citrate cycle (TCA cycle), photosynthesis, carbon metabolism, the biosynthesis of antibiotics, metabolic pathways, oxidative phosphorylation and the ribosome (Fig 2B). In addition, less than 10% of proteins were identified as differentially expressed proteins corresponding to the TCA cycle, photosynthesis, carbon metabolism and oxidative phosphorylation.

### Cluster of differentially expressed proteins

**Fig 3:**
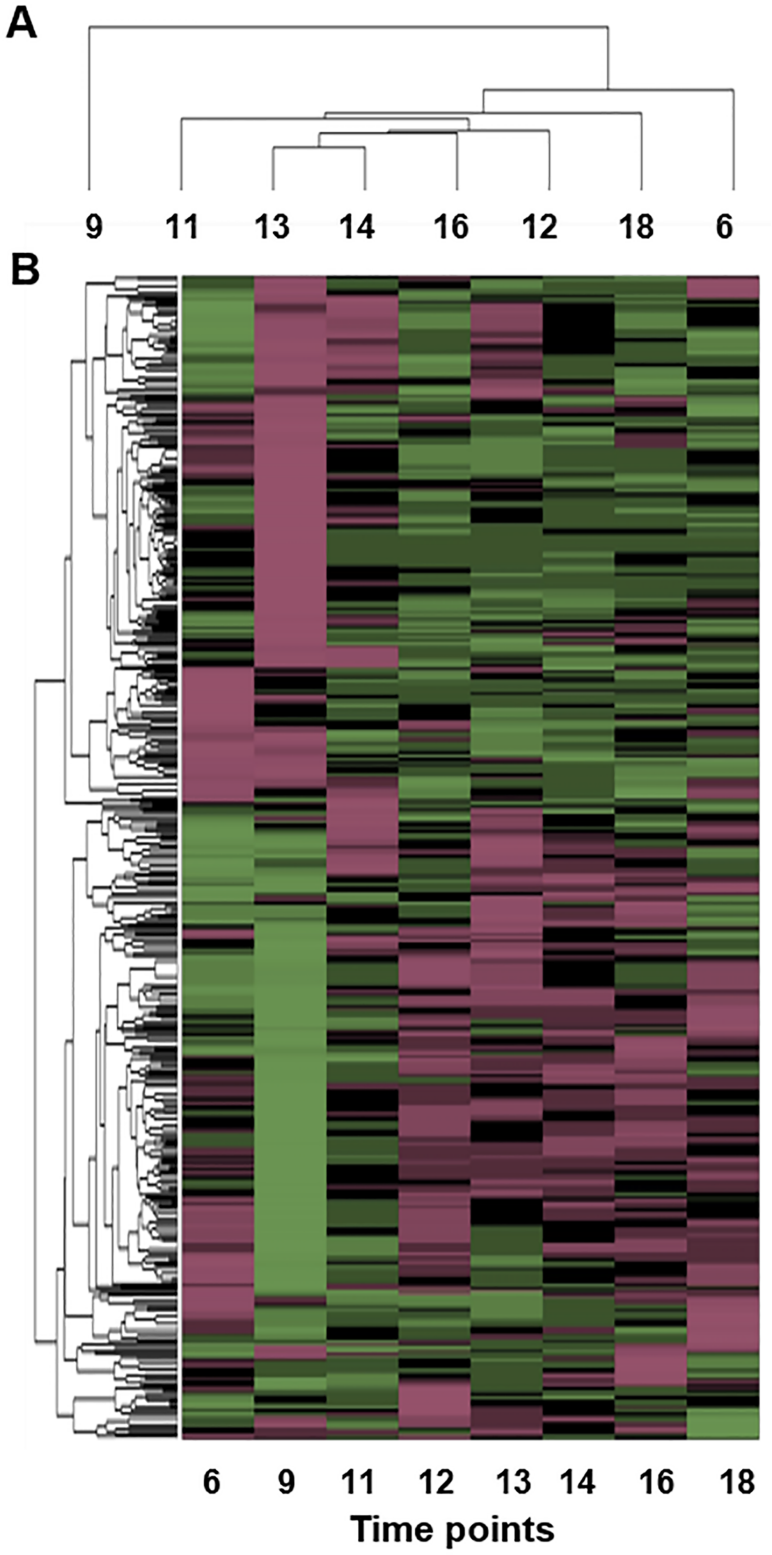
Dendrogram heat map of differential protein levels. (A) Clustering of proteomics data based on time points. Note that the data corresponding to 9 o’clock contribute major differences. (B) Clustering of proteomics data based on differential proteins.

The dynamic expression levels of the proteins at eight diurnal time points were quantitatively compared to those measured at the first time point. A clustering analysis of differentially expressed proteins was conducted with hierarchical clustering using the Pearson correlation as a distance metric; the resulting dendrogram is shown in Fig 3. These proteins can be classified into two major groups, depending on their behaviour at the second time point: 9 AM (Fig 3A). The subgroups were primarily defined by the expression level at the first and last time points (i.e., 6 AM; Fig 3A). The expression levels at other time points were diverse and contributed to achieving a more detailed classification. The differential protein levels were primarily clustered into two classes according to their level at the 6 AM and 9 AM time points (Fig 3B).

**Fig 4:**
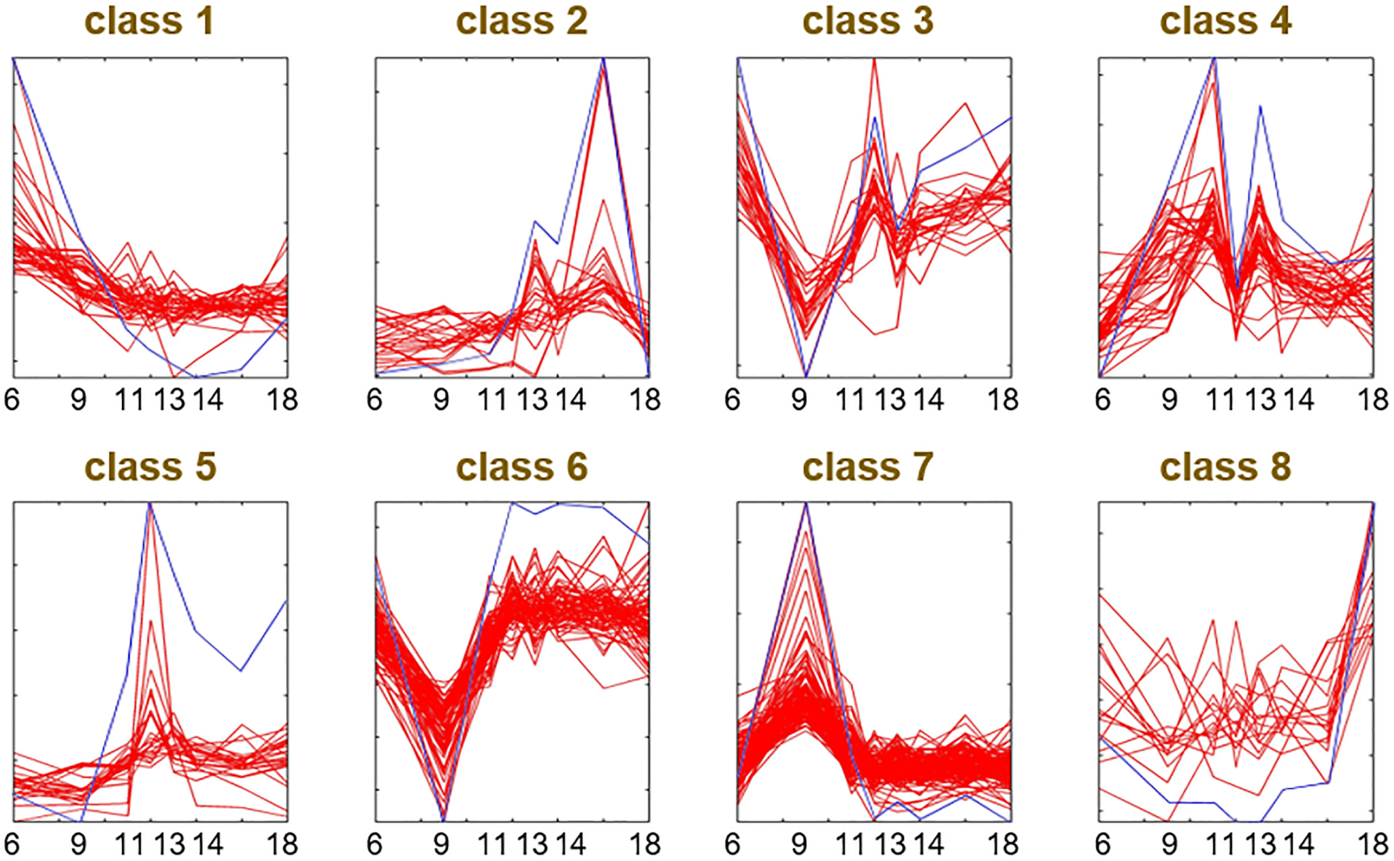
The k-means clustering of differentially expressed proteins. Blue lines indicate the centroid of the k-means in each group. Red lines indicate the dynamic patterns level of proteins in each group. x-axis is the sampling time points used for proteomics analysis.

These proteins were also classified into eight groups by k-means clustering based on their dynamic protein levels (Fig 4 and S4 Table). As shown in Fig 4, the k-means centroid displayed the typical expression pattern for each group. In general, the protein levels in classes 2, 4, 5 and 7 exhibited peak-like patterns, especially class 4, which was double peaked. Additionally, the proteins in class 5 showed a high peak at 12 o’clock. The patterns of classes 1 and 3 had downward trends, whereas the proteins in classes 6 and 8 increased from 6 AM to 6 PM. Finally, classes 3 and 6 exhibited level valleys at 9 AM.

Functional enrichment analysis was performed to determine the general relationship between protein function and expression pattern (S3 Table). In class 1, proteins were enriched in the structural constituents of the ribosome and translation. In class 2, proteins were enriched in protein processing in the endoplasmic reticulum. In class 4, proteins were enriched in carbon metabolism. In class 5, proteins were enriched in translation elongation factor activity, GTPase activity, GTP binding and RNA transport. In class 6, proteins were enriched in protein folding. In class 7, proteins were enriched in metabolic pathways, photosynthesis, the TCA cycle, and the biosynthesis of secondary metabolites and antibiotics. However, this enrichment was not significant, with large corrected p-values (10E-04) in other classes.

### Photosynthesis-related proteins

To understand the dynamic expression patterns of maize proteins involved in the photosynthesis pathway and carbon fixation, the relevant proteins were selected for further analysis.

#### Photosynthesis

Forty identified proteins were members of a photosystem that includes 101 proteins in the maize genome according to KEGG annotation; 24 were identified in this study. These proteins were involved in most of the key protein complexes of photosynthesis, such as Photosystems I and II, cytochrome b6/f, photosynthetic electron transport and F-type ATPase. Seventeen proteins displayed differential expression from 6:00 to 18:00 (i.e., during the light period of the diurnal cycle) (Table 1). Half of the proteins involved in the photosynthesis complexes, including Photosystems I and II, cytochrome b6/f and photosynthetic electron transport, showed differential levels. Only members of the F-type ATPase complex showed a steady pattern. Among the differentially expressed proteins related to photosynthesis, oxygen-evolving enhancer protein 1 belongs to class 1, which decreased from 6 AM to 6 PM. In contrast, oxygen-evolving enhancer protein 3–1 and ATP synthase delta chain are in class 3, which shows a double valley-like pattern at 9 o’clock and 13 o’clock. PSII 43 kDa protein (psbC) belongs to class 4 because it shows a double-peak pattern, whereas ferredoxin-like protein is in class 6 and has a valley-like pattern at 9 o’clock. Oxygen-evolving enhancer protein 3, photosystem I reaction centre subunit III, photosystem II 22 kDa protein, PSI P700 apoprotein A2 (psaB), photosystem II protein D1 (psbA), photosystem II protein D2 (psbD), ATPase subunit I (atpF) and photosystem II subunit PsbS1 (psbs1) belong to class 7 and exhibited a peak pattern at 9 o’clock. The remaining identified proteins in this pathway have steady levels from 6 AM to 6 PM.

**Table 1:** Proteins with differential levels during diurnal light cycle that are related to photosynthesis and carbon metabolism. The class of each protein was k-means clustered based on the dynamic protein levels measured at eight time points.

KEGG Pathway	Accession	Gene Name	Class	Coverage	# Unique Peptides	# AAs	MW [kDa]	Calc. pI
Photosynthesis	B4FRF2	oxygen-evolving enhancer protein 1	1	32.17	2	230	24.47	5.02
	B6T3B2	oxygen-evolving enhancer protein 1	1	25.53	2	329	34.50	5.64
	B4F9R9	uncharacterized LOC100191684	3	20.60	2	335	35.06	5.66
	B4G259	oxygen-evolving enhancer protein 3–1, chloroplastic	3	7.37	2	217	23.12	9.77
	B6SRD4	oxygen-evolving enhancer protein 3–1, chloroplastic-like	3	7.55	2	212	22.67	9.8
	B6T0C6	ATP synthase delta chain	3	8.00	2	250	26.66	4.78
	C0P6Z6	ATP synthase delta chain	3	8.00	2	250	26.70	4.78
	P48187	PSII 43 kDa protein (psbC)	4	6.55	3	473	51.87	7.03
	B6SP61	ferredoxin-like	6	25.81	4	155	16.14	5.85
	B4FT19	oxygen-evolving enhancer protein 3	7	7.79	2	231	25.33	7.8
	B6SRI3	photosystem I reaction center subunit III	7	8.00	2	225	23.79	9.36
	B6TKD1	photosystem II 22 kDa protein	7	9.96	3	271	28.36	9.09
	P04967	PSI P700 apoprotein A2 (psaB)	7	3.54	2	735	82.61	7.15
	P48183	photosystem II protein D1 (psbA)	7	6.80	2	353	38.99	5.36
	P48184	photosystem II protein D2 (psbD)	7	6.52	2	353	39.58	5.95
	P48186	ATPase subunit I (atpF)	7	13.66	3	183	20.97	9.23
	Q6WFB1	photosystem II subunit PsbS1 (psbs1)	7	10.19	3	265	27.70	9.09
Carbon fixation in photosynthetic organisms	C0P3W9	phosphoenolpyruvate carboxykinase	1	6.45	4	667	73.40	6.93
	Q9SLZ0	phosphoenolpyruvate carboxykinase	1	4.35	3	666	73.27	7.03
	P00874	rbcL	4	21.01	10	476	52.67	6.8
	P04711	pep1	4	23.09	20	970	#####	6.1
	Q43267	pep1	4	24.12	21	970	#####	6.04
	B6SLL8	malate dehydrogenase, cytoplasmic	6	5.12	2	332	35.50	6.09
	C4IZW9	mdh5	6	18.37	6	332	35.57	6.09
	B4G1J6	uncharacterized LOC100274515	7	20.61	2	131	13.82	8.97
	B6STH5	phosphoglycerate kinase	7	16.67	7	480	49.84	6.46
Starch and sucrose metabolism	Q8L5G8	uncharacterized LOC542510	1	6.29	3	509	54.78	6.49
	B4FQC6	uncharacterized LOC100382805	4	2.59	2	617	67.50	5.99
	B6SWT4	glucose-6-phosphate isomerase	4	2.60	2	616	67.47	5.99
	C0PAU7	uncharacterized LOC100382805	4	2.60	2	616	67.39	5.81
	Q8L5H0	sus2	4	3.34	3	809	91.87	6.61
	B5AMJ8	uncharacterized LOC100285259	7	4.53	3	838	94.39	7.28
	B6TCZ8	AGP2	7	4.63	2	518	57.41	6.83
	O48900	gss1	7	3.44	2	698	75.50	6.39
	P31927	sps1	7	7.21	6	1068	#####	6.67
Citrate cycle (TCA cycle)	B4F8B8	uncharacterized LOC100272834	1	5.57	2	341	37.52	6.33
	B6TS21	succinyl-CoA ligase beta-chain	1	26.54	3	422	45.17	6.32
	C0P3W W9	phosphoenolpyruvate carboxykinase (LOC541622)	1	6.45	4	667	73.40	6.93
	Q9SLZ0	phosphoenolpyruvate carboxykinase (LOC541622)	1	4.35	3	666	73.27	7.03
	C0PD24	uncharacterized LOC100383847	4	7.69	3	416	47.73	6.32
	C0PDA6	uncharacterized LOC100279871	5	4.82	2	498	53.55	7.42
	B6SLL8	malate dehydrogenase, cytoplasmic	6	5.12	2	332	35.50	6.09
	C4IZW9	mdh5	6	18.37	6	332	35.57	6.09
	Q9ZQY3	pyruvate dehydrogenase 2 (pdh2)	6	5.90	2	373	39.79	5.76
	B4F9M9	uncharacterized LOC100191657	7	12.75	2	400	45.40	6.39
	B4G249	uncharacterized LOC100274592 (gpm541)	7	14.08	3	412	46.19	6.55
	B6SRL2	aconitase 2 (aco2)	7	6.85	4	905	98.88	6.48
	B6TD54	uncharacterized LOC100282948	7	4.50	2	422	46.61	5.72
	B6TQ36	pyruvate dehydrogenase E1 component subunit beta	7	5.05	2	396	42.29	7.02
	C0HER4	uncharacterized LOC100304315	7	10.07	2	685	74.27	6.13
	C0PFT6	uncharacterized LOC100383412	7	3.07	2	814	92.22	7.18

#### Carbon fixation in photosynthetic organisms

Fifty identified proteins were enzymes involved in carbon fixation in photosynthetic organisms, and 111 annotated proteins were found in the maize genome (S4 Table). These proteins covered most pathways involved in carbon fixation, including the C4-dicarboxylic acid cycle, crassulacean acid metabolism (CAM), and the reductive pentose phosphate cycle (sucrose synthesis). Nine proteins were differentially expressed: phosphoenolpyruvate carboxylase 4 (4.1.1.31) [38, 39], malate dehydrogenase (1.1.1.40) [40] in the C4-dicarboxylic acid cycle, phosphoenolpyruvate carboxylase 1/4 (pep1/4) (4.1.1.31) and malate dehydrogenase (1.1.1.40) in the CAM pathway, phosphoglycerate kinase (2.7.2.3) [41], and the Rubisco large subunit (rbcL) (4.1.1.39) [42, 43]. According to their expression patterns, phosphoenolpyruvate carboxykinase belongs to class 1 because it exhibits a decreasing pattern. rbcL and pep1 were clustered into class 4 (showing double peaks at 11 AM and 1 PM). Malate dehydrogenase, cytoplasmic, and malate dehydrogenase 5 (mdh5) are in class 6 (a valley-like pattern at 9 AM). Phosphoglycerate kinase, which showed a peak pattern at 9 AM, belongs to class 7.

#### Starch and sucrose metabolism

Hexokinase, glucose-6-phosphate isomerase, sucrose synthase 2 (sus2), ADP-glucose pyrophosphorylase (AGP2), starch synthase homologue 1 (gss1) and sucrose phosphate synthase 1 (sps1) were identified as having differential protein levels in starch and sucrose metabolism and were clustered into either class 4 or 7 according to their protein levels.

#### Citrate cycle

Thirty identified proteins were enzymes belonging to the citrate cycle (TCA), for which 96 proteins have been annotated in maize (S4 Table). Differential expression was found for 15 proteins, including citrate synthase (2.3.3.1) [44], malate dehydrogenase, cyto, mdh5, aconitate hydratase (4.2.1.3), phosphoenolpyruvate carboxykinase, isocitrate dehydrogenase (NAD+) (1.1.1.41) [45, 46], the succinyl-CoA synthetase beta subunit (6.2.1.4/6.2.1.5) [47, 48] and the pyruvate dehydrogenase E1 component alpha/beta subunit (1.2.4.1) [49, 50]. Succinyl-CoA ligase and phosphoenolpyruvate carboxykinase showed decreased levels and belong to class 1. Malate dehydrogenase and pyruvate dehydrogenase E1 beta subunit isoform 1 are in class 6. Isocitrate dehydrogenase, aconitate hydratase, cytoplasmic, ATP-citrate synthase and pyruvate dehydrogenase E1 component subunit beta are clustered in class 7.

### Protein profile validation by qRT-PCR

**Fig 5:**
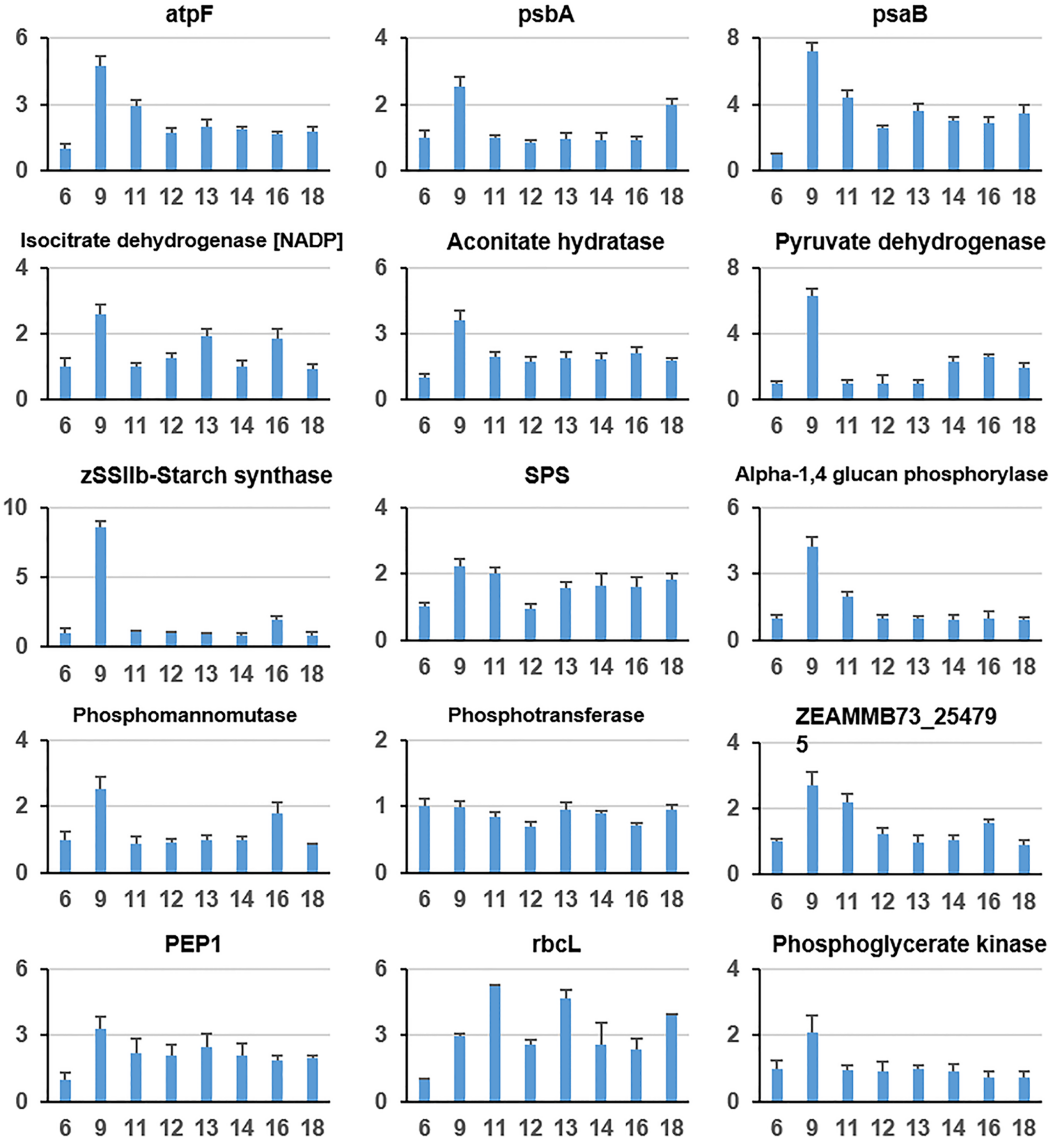
Expression levels of 15 selected genes that are related to photosynthesis and carbon metabolism. The expression levels of each gene are presented relative to the first time point (i.e., 6 AM). Values are the means ±standard error (SE) of three independent experiments with at least three replicates each.

qRT-PCR was further applied to validate the quality of the proteomics. Fifteen genes related to photosynthesis and carbon metabolism were selected for quantification (Fig 5). The expression patterns of these genes were consistent with iTRAQ-labelled proteomics data. For instance, *atpF* gene expression shows a peak-like pattern with a maximum at 9 o’clock, whereas the dynamic pattern of atpF protein level corresponds to class 7, which also exhibits a peak-like pattern with a maximum at 9 o’clock. The other genes shown in Fig 5 have similar expression and protein level patterns. This result indicated that the quality of the proteomics data is acceptable.

## Discussion

Leaves are the key organ in plants in which photosynthesis occurs, which supplies energy and carbon materials. One important regulatory mechanism of photosynthesis is the control of enzyme protein levels. During a diurnal cycle with varying light intensity, leaves optimize their photosynthesis rates to adapt to the environment by adjusting enzyme levels [51].

Maize is a crop that exhibits highly efficient photosynthesis and a one-peak rate pattern. Plants with one-peak photosynthesis rate patterns can open more stomata and suffer less photoinhibition than crops with two-peak rate patterns after midday, when the light intensity reaches its maximum [18]. In this study, we identified approximately 1,749 proteins with different dynamic patterns from 6 AM to 6 PM. Among them, several proteins are involved in metabolic pathways. The results suggest the existence of a mechanism by which maize leaves control their photosynthesis-related enzymes. In total, 1,126 identified proteins were steadily expressed during the light portion of the diurnal cycle. The differentially expressed proteins are involved in photosynthesis, carbon metabolism, and the citrate cycle. These are important pathways related to photosynthesis rates, and thus, maize leaves control the expression levels of photosynthesis-related proteins to alter the photosynthesis rate.

The differentially expressed proteins were primarily classified based on their expression patterns at 9 AM. Typically, the photosynthesis rate increases at approximately 6 AM and peaks at 9 AM. Plants with one-peak photosynthesis rate patterns can maintain a high photosynthesis rate until 4 PM. In contrast, plants with double-peak patterns exhibit reduced rates at 12 PM, which recover at approximately 4 PM. Therefore, 9 AM was used to cluster the expression patterns of the photosynthesis-related proteins. Regardless of whether a one- or two-peak pattern is shown, the primary goal of photosynthesis-related proteins is to adapt to increased light intensity. Many of these proteins have peak-like expression patterns, such as those in classes 2, 4, 5 and 7, and may be important for plant response to photoinhibition. The expression of proteins in classes 3 and 6 exhibits a double-peak pattern with a valley at midday, which suggests that the Leavenworth maize one-peak photosynthesis pattern has a reduced response to high light intensity at midday, similar to the double-peak pattern. Furthermore, the proteins in class 5, which show an expression peak at 12 PM, may contribute to the response to the maximum light intensity during the diurnal cycle.

Among the photosynthesis-related proteins identified in this study, photosynthesis antenna proteins remain at nearly steady levels throughout the diurnal cycle, indicating that light harvesting in chlorophyll does not change with varying light intensity. Indeed, the maize leaf does not adjust the photosynthesis antenna protein levels in response to changing light intensity. However, proteins in the photosystem display differential expression during the diurnal cycle. For example, Photosystems I and II have more variable expression than F-type ATPase. Furthermore, the proteins involved in Photosystems II and I exhibit reversed expression patterns relative to their levels at 9 AM, which suggests that maize leaves highly regulate the photosystem to adapt to variations in light intensity and thereby achieve efficient photosynthesis. In addition to photosynthesis, carbon fixation in leaves is one of the most important biological processes impacting the photosynthesis rate. Efficient carbon fixation benefits photosynthesis by reducing negative feedback. In plants, sucrose is the major product of carbon fixation from CO_2_. In this study, most of the enzymes involved in carbon fixation were identified, and their dynamic patterns were analysed. In general, the enzymes corresponding to each carbon fixation reaction step show dynamic expression in response to diurnal variations in light intensity. Intermediate metabolic production is the major regulator of this adjustment. In this study, in maize leaves, the lowest levels were observed at 9 AM, when the photosynthesis rate is high. This expression level was then maintained throughout the afternoon (i.e., from 12 PM to 6 PM). Maize is a C4 plant and performs the dicarboxylic acid cycle in its bundle sheath cells [18]. pep1/4 is the initial step of the dicarboxylic acid cycle to fix CO_2_ into oxaloacetate [38, 39]. Malate dehydrogenase is the final step to release CO_2_ [40]. Malate dehydrogenase exhibits a single-peak pattern with a maximum at 9 AM. In contrast, phosphoenolpyruvate carboxylase 4 has a double-peak pattern, which suggests that maize leaves increase the rate at which CO_2_ is fixed into oxaloacetate in the afternoon. The most efficient utilization of CO_2_ for sucrose synthesis occurs in the morning at 9 AM, when the level of malate dehydrogenase is maximized. In addition, most TCA cycle enzymes are dynamically regulated during the diurnal cycle. Isocitrate dehydrogenase, which is involved in the limiting step, is increased at 11 AM and subsequently maintains a steady level throughout the afternoon. However, whether this dynamic pattern is beneficial for optimizing the photosynthesis level remains unknown.

In summary, this study revealed the expression patterns of important metabolic enzymes related to photosynthesis. Maize leaf maintains some proteins at steady levels during the diurnal light cycle, whereas those involved in photosynthesis, carbon fixation and the TCA cycle are highly dynamically regulated and adapt to diurnal variations in the light intensity. Despite the overall one-peak photosynthesis rate pattern of maize during the diurnal cycle, some related enzymes exhibit double-peak expression patterns. Typically, expression peaks at 12 AM, 1 PM and 2 PM suggest the existence of a regulatory mechanism in response to high light intensity at midday.

## Datasets

The raw data accompanying this study have been deposited in iProX with the identifier IPX00085001.

## Supporting information

S1 Table Sequences of the primers used for real-time PCR. (XLSX)

S2 Table Total numbers of proteins identified by proteomics analysis. (XLSX)

S3 Table Proteins exhibiting differential levels and k-means clustering. (XLSX)

S4 Table Raw data analysis. (XLSX)
